# Supplementary material for: High-risk fertility behaviors and associated factors among married reproductive-age women in sub-Saharan Africa: A multilevel mixed-effect analysis of nationally representative data from 35 countries
Source: PLOS Glob Public Health. 2024 Sep 13;4(9):e0003446. doi: 10.1371/journal.pgph.0003446 (PMC11398670; doi:10.1371/journal.pgph.0003446)
Supplement: Attachment — Submitted filename: Response to Reviewers.docx [file pgph.0003446.s001.docx]

**Dear Academic Editor,**

Thank you for the opportunity to revise our manuscript “***High-risk fertility behaviors and associated factors among married reproductive-age women in sub-Saharan Africa: a multilevel mixed-effect analysis of nationally representative data from 35 countries (Manuscript ID: PGPH-D-24-00717).***”. We have addressed the concerns raised by the reviewers using a point-by-point response as stated below. The amendments made to the manuscript have been presented using track change in the attachment titled “Revised Manuscript with Track Changes”.

**Response to Editor’s comments**

**Journal Requirements**:

**Comment 1:** Please provide separate figure files in .tif or .eps format only and remove any figures embedded in your manuscript file. Please also ensure all files are under our size limit of 10MB.

**Response 1:** Thank you. We have provided all figure files in .tif format and ensured that all figure files adhere to the size limit stated in the journal requirement.

**Comment 1:** We noticed that you used "unpublished" in the manuscript. We do not allow these references, as the PLOS data access policy requires that all data be either published with the manuscript or made available in a publicly accessible database. Please amend the supplementary material to include the referenced data or remove the references.

**Response 2:** Thank you very much for your important recommendation. We have removed the stated reference as suggested.

**Response to Reviewer 1 Comments**

Overall, the article provides a comprehensive analysis of high-risk fertility behaviors among married women in sub-Saharan African countries, utilizing data from 35 countries and employing advanced statistical techniques. This study is unique enough and more convincing timely output of the topic covered. The study sheds light on both individual and community-level factors associated with the risk of fertility, offering valuable insights for policymakers and public health practitioners. However, there are several points that could enhance the clarity, robustness, and impact of the research:

**Comment 1**: Methods (page 6): the methodology section seems to be strong enough however, it outlines the use of multivariable multilevel logistic regression to analyze determinants of high-risk fertility behaviors, more detailed information on model selection criteria and potential confounders could strengthen the methodological rigor. Providing justification for the choice of specific variables and discussing potential biases would further enhance the validity of the findings.

**Response 1**: Thank you for your interesting suggestion. Information regarding model selection criteria, potential confounders, and variable selection was provided under the “Variables” and “Data Management and Statistical Analysis” sections of the revised manuscript (pages 7-10). Additionally, we have supported the choice of variables with citations in the revised manuscript.

**Comment 2**: Methods (page 6): there has been a potential sampling bias as given the cross-sectional nature of the data and the possibility of recall bias. Providing information on the representativeness of the sample and efforts taken to ensure data reliability could support the credibility of the study findings. Also, what mechanism did you take to minimize the bias is to be reported.

**Response 2**: Thank you again for your important recommendation. We agree that there is a potential sampling and recall bias in the DHS survey. Thus, we have applied a sample weight procedure to compensate for the non-representativeness of the sample as stated in the main manuscript. Moreover, since we used secondary data, it’s impossible to manage the problem associated with recall bias. For this reason, we acknowledged the potential of recall bias in the limitation section of the manuscript.

**Comment 3**: Results (page 10): the article presents a wealth of data on high-risk fertility behaviors across various sociodemographic and reproductive characteristics. However, a deeper exploration of the implications of these findings for different population (as categorized) subgroups would enrich the discussion. For instance, discussing the differential impact of interventions based on age, education, and geographic sub-location or based on the outcome variable could provide valuable insights for targeted public health strategies.

**Response 3**: Thank you for your recommendation. We have tried to include this concern under the “Policy implications” section of the revised manuscript.

**Comment 4**: the authors can add a section or sub-section under the discussion in the name of Policy Implications. While the discussion touches upon the importance of addressing modifiable risk factors and strengthening policies, further elaboration on specific policy recommendations and their feasibility within the context of sub-Saharan African countries would be beneficial. Providing concrete suggestions for policymakers, such as investments in education and family planning services or interventions at the community level, could enhance the practical relevance of the study.

**Response 4**: Thank you very much. We have provided the “Policy implications” subsection as suggested in the revised manuscript.

**Comment 5**: you can provide an outline of future research directions guided by the study findings and implications. Considering the complexity of factors influencing high-risk fertility behaviors, it would be valuable to propose avenues for future research. Exploring emerging trends, such as the impact of digital health interventions or cultural shifts, could offer new perspectives on addressing fertility-related challenges in the region, or whatever the authors think of appropriate based on the study outcome.

**Response 5:** Thank you very much for the nice suggestion. We have provided an outline of future research direction under the “Policy implications” and “Conclusion” sections of the revised manuscript.

**Response to Reviewer 2 Comments**

Authors have studied “High-risk fertility behaviors and associated factors among married reproductive-age women in sub-Saharan Africa: a multilevel mixed-effect analysis of nationally representative data from 35 countries”. The present work is a nice piece of work. I can give the recommendation for publication but before this, some minor revisions need to be addressed.

**Comment 1**: What is the advantage of this study? Write this in the introduction section and in the conclusion.

**Response:** Thank you very much. We have added the advantage of the study as suggested in the revised manuscript.

**Comment 2**: The research gap should be clearly written.

**Response 2**: Thank you for your interesting comment. We have provided the research gap in the background section of the revised manuscript.

**Comment 3**: Cite the properly the used equations.

**Response 3**: Thank you. We have properly cited the equations used in the manuscript.

**Comment** 4: Improve the discussion section for the graphs presented in the current work.

**Response 4**: Thank you for your interesting suggestion. We have incorporated the results presented in graphs in to discussion section of the revised manuscript.

**Comment 5:** Improve the conclusion and abstract.

**Response 5**: Thank you very much. We have modified the conclusion and abstract section accordingly.

**Comment 6:** What are the limitations of the present work and what are the future recommendations. Please mention them in the abstract.

**Response 6**: Thank you for your suggestions. We have provided the limitations of the study under the “Strengths and limitations” section of the manuscript and stated future recommendations in the conclusion section of the abstract and main body of the revised manuscript.

**Comment 7**: Check the entire manuscript regarding grammar and spelling mistakes.

**Response:** Thank you very much. We have thoroughly checked the manuscript for grammar and spelling errors.

**Response to Reviewer 3 Comments**

In the paper, the authors have analyzed the pooled prevalence of high-risk fertility behaviors and associated factors among married women in sub-Saharan African countries. I found this paper quite interesting and well-managed. To further improve this study, I suggest the following:
**Comment 1:** Authors should also include "Religion" as an explanatory variable as, for this study, it can play an important role and make the study more comprehensive.

**Response 1**: Thank you for your interesting comment and we agree with your concern. We have included religion as one of the explanatory variables considered for analysis, however, since there were differences in religious affiliations across the countries (i.e. the classification of the religion is not uniform across the included countries), we were not able to categorize them due to the inconsistences (see <https://www.idhsdata.org/idhs-action/variables/RELIGION#codes section> ). Thus, we finally decided to exclude this variable from the analysis.
